# Not Enough Natural Data? Sequence and Ye Shall Find

**DOI:** 10.3389/fmolb.2020.00065

**Published:** 2020-04-21

**Authors:** Arthur M. Lesk

**Affiliations:** Department of Biochemistry and Molecular Biology, The Pennsylvania State University, University Park, PA, United States

**Keywords:** correlated mutations, structure prediction, *in silicio* evolution, neighboring residues, high-throughput sequencing

Knowing pairs of residues in a protein that are close together in space, even if—*especially if* —they are not close together in the amino-acid sequence, allows determination of the three-dimensional structure. This was first shown by NMR protein structure determinations, in which the Nuclear Overhauser effect spectroscopy (NOESY) identifies pairs of protons in spatial proximity. Similar information is available from amino-acid sequence data if a family of homologous proteins shows correlated mutations in pairs of residues.

The underlying mathematics leading from contact information to three-dimensional structure tells us that, for any set of points, knowledge of the numerical value of the distance between every pair of points straightforwardly determines the three-dimensional structure (up to enantiomorph ambiguity). But neither NMR nor sequence analysis specifies the value of the distances precisely, and the information is limited to a small subset of pairs of atoms. Yet, by adding stereochemical constraints and energy functions, it has been possible to determine structures from limited neighbor data.

The approach via amino-acid sequence analysis is to identify pairs of positions that show *correlated mutations*. Correlated mutations are patterns of amino-acid substitutions appearing in multiple sequence alignments, where change in an amino acid at some position corresponds to change in the amino acid at one or more other positions. For the sequences in which the amino acid at the first position is constant, there is no mutation at the other positions also. Thus, in a set of positions showing strictly correlated mutations, each amino acid observed at every position corresponds to a unique amino acid at the other positions.

Why should correlated mutations appear? If the amino-acid sequence of a protein is tuned by selection to achieve a precise structure and function, a mutation may perturb these features. Mutations also, of course, allow proteins to explore neighborhoods in sequence space, to alter function—including for instance substrate specificity—and even to develop new functions (see The Structure-Function Linkage Database sfld.rbvi.ucsf.edu). However, a random succession of mutations would be expected to destroy function and even structure, as we see in pseudogenes. Selection for correlated mutations, then, is the “guard rail” that keeps the evolving proteins functional.

In many cases, a second mutation is compensatory—that is, it has the effect of repairing the insult to the protein from the first mutation. Often the structural compensation is *local*, that is, the amino acids corresponding to the two mutations are in contact. For this reason, correlated mutations generally indicate spatial proximity of the residues involved.

The idea of using correlated mutations to detect spatial proximity is an old one. A serious problem has been “transitivity”: If residue A is near residue B, and residue B is near residue C, mutations in A and C may appear to be correlated. A and C may be spuriously indicated as neighbors. What is necessary has been the development of methods to extract from the data the true neighbors: A and B and B and C.

Recognition of the problem led to its solution. What is necessary is to compute *partial correlations;* that is, correlations between each individual pair of residues after subtracting out the dependence on all the other variables. Suppressing these dependencies can eliminate, or at least reduce substantially, the transitivity effects. Use of partial correlations, plus calibrating the number of expected contacts to what is empirically observed in known protein structures of suitable size, improved the accuracy of inference of residues in contact from correlated mutations. A seminal paper was by Marks et al. (2011).

Analysis of correlated mutations has produced impressive results in the prediction of the structures of single proteins (e.g., Kosciolek and Jones, 2014), and also protein-protein complexes (Hopf et al., 2014) and RNAs (Weinreb et al., 2016). Correlated mutations play important roles in the leading protein structure prediction projects, as assessed in the most recent CASP programme (Zheng et al., 2019); these include Rosetta (Ovchinnikov et al., 2016), and AlphaFold (Senior et al., 2019).

Mutations affect dynamics as well as structure; a very interesting dimension of protein evolution. Butler et al. (2018) have studied the effect of correlated mutations on atomic motions. In principle, correlated mutations that affected function but not structure, via changes in dynamics, would be missed in a protocol selective for function. However, success of applications of correlated mutations to protein structure prediction suggests adequate persistence of signal.

## 1. But one needs the sequences …

To achieve statistically-meaningful results, thousands of sequences are required. Widespread genomic sequencing has produced copious amounts of amino-acid sequence information. Indeed, for many families of proteins, enough data are available to allow detection and application of correlated mutations. But what if adequate sequence information is not available? For example, a protein with limited species distribution, or a recently-evolved protein, or even a *de novo* designed protein?

In such cases, why not “roll your own” (sequences)? Two techniques support the *in vitro* extension of the correlated-mutation approach. These are the ease of creating mutant proteins by error-prone PCR (Wilson and Keefe, 2001), and the ability to select from among the mutants those that retain function of a selected protein. Recently, several papers have successfully applied these ideas to generate mutated proteins, and to use correlated mutations in their sequences to determine neighbors (Fantini et al., 2019; Rollins et al., 2019; Schmiedel and Lehner, 2019; Stiffler et al., 2019).

Work reported in recent papers has demonstrated that these methods could determine neighboring residues in the B1 domain of Streptococcal protein G (Schmiedel and Lehner, 2019), or in β–lactamases (bacterial proteins that cleave the β–lactam ring of penicillin, conferring resistance) (Fantini et al., 2019; Stiffler et al., 2019), and acetyltransferase AAC6 (Stiffler et al., 2019).

## 2. Too few sequences? Get over the hump with *CAMELS*: *Coupling Analysis by Molecular Evolution Library Sequencing*

Using methods similar to those of directed evolution (Arnold, 2018, 2019), Fantini et al. (2019) subjected a β−lactamase-encoding plasmid to alternating cycles of mutation, by error-prone PCR; and selection, by survival when challenged by a medium containing the β−lactamase inhibitor ampicillin. In each “generation” plasmids were sequenced using the Pacific Biosciences Sequel platform, using the SMRT (Single Molecule, Real Time) method. It was necessary to use a sequencing method that not only had adequate capacity to deal with the large number of mutants, but one for which the accuracy was constant over a read length longer than the gene.

The challenge in design of the protocol is to achieve a high mutational load but retain an adequate survival rate, while keeping the number of transformants within the sequencing capacity of the Sequel platform. The number of mutants increased approximately linearly in successive generations of mutation/selection cycles. After twelve generations, the sequences had a median of 25 mutations of amino acids per protein (not nucleotides per gene, which would include silent mutations). This is approximately 10% of the length of the protein.

The sequence data produced were treated by computational methods similar to those proven successful for natural sets of homologous sequences.

The results presented by Fantini et al. provide proof of principle. That a predicted structure of TEM-1 β−lactamase does not appear is attributable to the distribution of pairs of positions in contact: The distance map produced shows short and medium range distances (measured as distance between the positions of the amino acids in the sequence). However, compared to the natural evolutionary data set, from Uniprot, it lacks the long-range distances necessary to build a three-dimensional structure. It is likely that pushing the technique will overcome this problem.

Another apparent limitation is the necessity for a method for selection. In this respect, TEM-1 β−lactamase has ideal properties. But many proteins will not. It may be that protein interactions more general than enzyme-substrate interactions will come to the rescue.

Stiffler et al. (2019) applied similar methods to two other proteins: aminoglycoside acetyltransferase AAC6 (130 residues) and β−lactamase PSE1 (266 residues).

## 3. Prediction of the Structures

Rollins et al. (2019) and Schmiedel and Lehner (2019) carried out the second part of the proof of principle. Using the dataset of Olson et al. (2014), who performed extensive mutagenesis and functional evaluation of the GB1 domain, they predicted the structure of the B1 Immunoglobulin-binding domain of Streptococcal protein G, a 56-residue domain. Olson et al. (2014) performed the *allumwandlung*^1^ (= replacement of the natural residue with all 19 other possibilities), at all positions except for the N-terminal methionine, producing 55 × 19 = 1, 045 single mutants. Olson et al. (2014) also produced 535917 of the possible 55 × 54/2 × 19 = 536085 double mutants. Selection was for binding to an IgG fragment.

For the best structure predictions of GB1 by Rollins et al. (2019) and by Schmiedel and Lehner (2019), based on this dataset, the r.m.s.d. of all Cα atoms between the predicted structures and the experimental structure (wwPDB codes 1PGA and 1PGB) were in the range of 1.9−3 Å.

Stiffler et al. (2019) derived from their mutated sequence data the three-dimensional structures of aminoglycoside acetyltransferase AAC6 and β−lactamase PSE1. The predictions closest to the experimental structures had r.m.s.d.'s of 4.5 Å for 240 out of 266 residues for PSE1 and 3.8 Å for 122 out of 130 residues for AAA6.

It should not be overlooked that these are predictions of *known* structures, unlike the blind tests of the CASP programmes. There is no reason whatsoever to think that the authors “peeked” at the answer. However, the sophistication and complexity of learning algorithms trained on the wwPDB—as used by Schmiedel and Lehner (2019), but *not* by Fantini et al. (2019), Rollins et al. (2019), nor Stiffler et al. (2019)—can make the contributions of these methods sufficiently obscure that it may be difficult to exclude the inadvertent “leakage” of information about the target structure into the prediction.

There is an obvious question about how the method will scale up to larger proteins (Chiasson and Fowler, 2019 call attention to this point). After all, the number of possible pairs of positions goes as the square of the sequence length. Rollins et al. (2019) suggest that structures of comparable quality could be computed from only small fraction of the contacts [this was also explored some time ago by Skolnick et al. (1997), and Kolinski and Skolnick (1998)]. Neverless a fractional decrease in the number of contacts is still only a linear advantage fighting against the quadratic increase in the potential data.

## 4. Conclusion

The results appearing in these recent publications are an essential step toward the establishment of another powerful approach to structure determination of biological macromolecules and complexes. If not the beginning of the end, they are at least the end of the beginning.

## Author Contributions

The author confirms being the sole contributor of this work and has approved it for publication.

## Conflict of Interest

The author declares that the research was conducted in the absence of any commercial or financial relationships that could be construed as a potential conflict of interest.

## Footnotes

^1^The *allumwandlung* is a chess problem in which, in different variations, a pawn is promoted to all possible other pieces: knight, bishop, rook, queen.
